# Ochratoxin A Induces Oxidative Stress in HepG2 Cells by Impairing the Gene Expression of Antioxidant Enzymes

**DOI:** 10.3390/toxins13040271

**Published:** 2021-04-09

**Authors:** Enrique García-Pérez, Dojin Ryu, Chan Lee, Hyun Jung Lee

**Affiliations:** 1 School of Food Science, Washington State University, P.O. Box 646376, Pullman, WA 99164-6376, USA; enrique.gape@gmail.com; 2 Department of Animal, Veterinary, and Food Sciences, University of Idaho, 875 Perimeter Drive MS 2330, Moscow, ID 83844-2330, USA; dryu@uidaho.edu; 3 Department of Food Science and Technology, Chung-Ang University, Anseong, Gyeonggi 17546, Korea

**Keywords:** ochratoxin A (OTA), hepatotoxicity, HepG2, oxidative stress, reactive oxygen species (ROS)

## Abstract

Ochratoxin A (OTA) is a mycotoxin frequently found in raw and processed foods. While it is considered a possible human carcinogen, the mechanism of action remains unclear. OTA has been shown to be hepatotoxic in both in vitro and in vivo models and oxidative stress may be one of the factors contributing to its toxicity. Hence, the effect of OTA on human hepatocellular carcinoma, HepG2 cells, was investigated on oxidative stress parameters. The cytotoxicity of OTA on HepG2 was time- and dose-dependent within a range between 0.1 and 10 µM; while 100 μM of OTA increased the intracellular reactive oxygen species (ROS) in a time-dependent manner. Additionally, the levels of glutathione (GSH) were increased by 9.7% and 11.3% at 10 and 100 nM of OTA, respectively; while OTA at 100 μM depleted GSH by 40.5% after 24 h exposure compared with the control. Finally, the mRNA level of catalase (CAT) was downregulated by 2.33-, 1.92-, and 1.82-fold after cells were treated with 1, 10, and 10 μM OTA for 24 h, respectively; which was linked to a decrease in CAT enzymatic activity. These results suggest that oxidative stress is involved in OTA-mediated toxicity in HepG2 cells.

## 1. Introduction

Ochratoxin A (OTA) is a fungal toxin produced by many species in the genera Aspergillus and Penicillium. Due to their diverse growth characteristics, OTA has been found in a wide variety of agricultural commodities and their processed products including major cereals, nuts, coffee, dried fruits, and wine [1,2,3,4,5]. In addition to its potent renal carcinogenic activity, OTA is known to be mutagenic, teratogenic, neurotoxic, immunotoxic, hepatotoxic, and embryotoxic in experimental animal models [6,7,8,9,10,11]. For these reasons, OTA represents a public health concern and has been classified as a potential human carcinogen in Group 2B by the International Agency for Research on Cancer (IARC) [12].

Until now, there is no consensus regarding its mechanisms of action, especially whether it acts through a genotoxic or non-genotoxic mechanism. Both in vitro and in vivo studies have shown that OTA toxicity may be related to the stimulation of oxidative stress as OTA increase levels of reactive oxygen species (ROS), oxidative DNA lesions, lipid and protein damage, and depletion of endogenous antioxidants such as glutathione and antioxidant enzymes [13,14,15,16,17]. Glutathione (GSH) is the most important cell redox potential buffering molecule as it controls ROS levels either directly (e.g., reaction with O_2_^•−^ or H_2_O_2_) or serves as an electron donor for ROS-utilizing enzymes such as glutathione peroxidase (GPX). Antioxidant enzymes include superoxide dismutase (SOD), catalase (CAT), and GPX. SOD converts O_2_^•−^ to molecular oxygen (O2) and H_2_O_2_, whereas CAT converts H_2_O_2_ into oxygen and water, and GPX converts H_2_O_2_ into water [18]. In addition, GPX requires glutathione reductase (GSR) and glucose-6-phosphate dehydrogenase (G6PD) to regenerate GSH and reduced nicotinamide adenine dinucleotide phosphate (NADPH) from its oxidized counterparts, glutathione disulfide (GSSG), and NADP^+^, respectively [18].

The liver is the most important organ involved in drug metabolism. Drugs may accumulate in the liver and/or enzymes in the liver may form toxic drug metabolites, leading to hepatotoxicity [19]. OTA has been shown to be hepatotoxic in both in vitro and in vivo studies; however, it is still not clear how OTA exerts its hepatotoxicity [6,7,8,9,10,11]. HepG2 cells are derived from human hepatocellular carcinoma and have been used in many toxicological studies despite their low levels of specific enzymes involved in phase I and phase II metabolism [19,20]. More importantly, HepG2 cells have been shown to be sensitive toward OTA as well as other xenobiotics, while ROS formation and GSH depletion are two important mechanisms of drug-induced hepatotoxicity [15,19,21,22,23]. Thus, this study aimed to examine whether OTA-mediated hepatotoxicity is related to an induction of oxidative stress in HepG2 cells.

## 2. Results

### 2.1. Cytotoxicity of Ochratoxin A (OTA)

**Figure 1:**
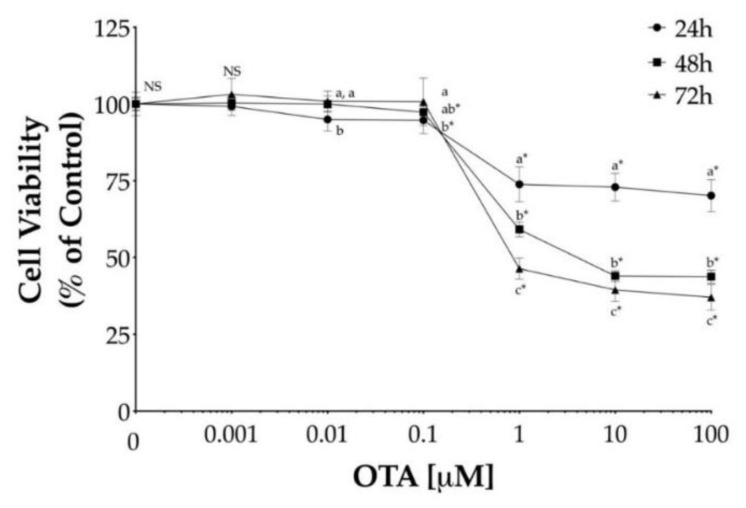
Cytotoxicity of ochratoxin A (OTA) in human hepatocellular carcinoma (HepG2) cells. Cell viability was determined by the reduction of 3-(4,5-Dimethyl-2-thiazolyl)-2,5-diphenyl-2H-tetrazolium bromide (MTT) after incubation with OTA for 24 h (●), 48 h (■), and 72 h (▲) in HepG2 cells. Values are mean ± standard deviation of three independent experiments and are expressed as percentage of control. Different letters indicate a significant difference (*p* < 0.05) between values within the same OTA concentration by Tukey’s multiple comparison test while ns = non-significant. Asterisks (*) indicate a significant difference (*p* < 0.05) between values against each group control by Dunnett’s test.

To determine the effect of OTA on cell viability, HepG2 cells were treated with OTA at varying concentrations, ranging from 1 nM to 100 μM for different time intervals (24, 48, and 72 h). In general, OTA led to a dose- and time-dependent decrease in cell viability while dose-dependent decrease plateaued above 1 μM, as shown in Figure 1. The lowest concentration of OTA that exhibited a significant difference (*p* < 0.05) in cell viability was 1 μM at each exposure time (Figure 1). However, the minimum levels of cell viability after 48 h and 72 h exposure to 10 μM OTA were 44% and 40%, respectively, and it did not decrease further while increasing OTA concentrations.

### 2.2. Effect of OTA on Reactive Oxygen Species (ROS)

**Figure 2:**
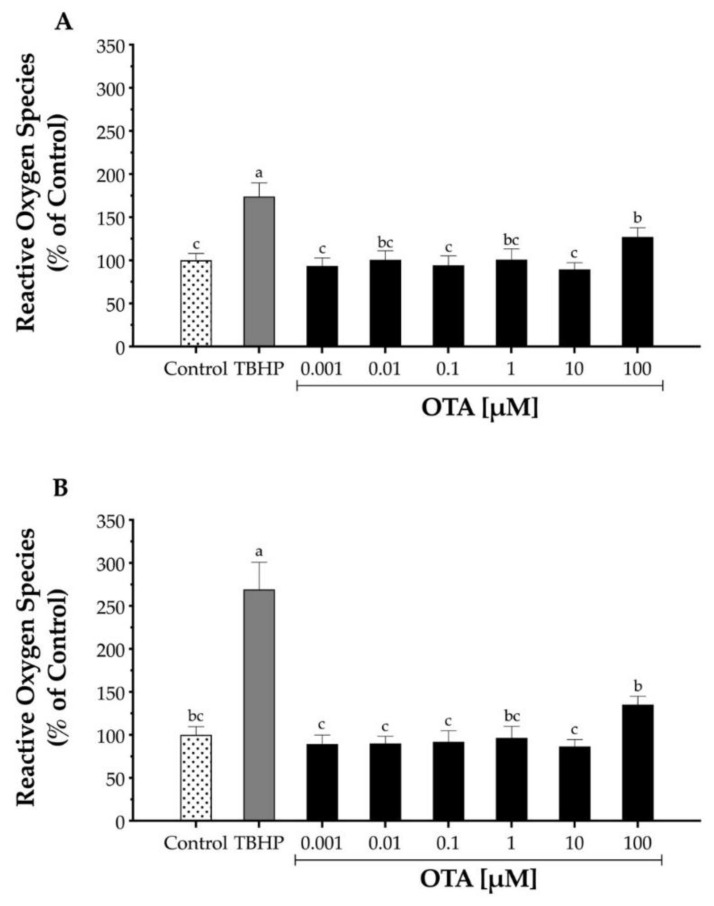
Reactive oxygen species production by *t*-butyl hydroperoxide (TBHP) and ochratoxin A (OTA) in human hepatocellular carcinoma (HepG2) cells. Increase in DCF fluorescence upon oxidation of carboxy-H2DCF was monitored after 4 h (**A**) and 24 h (**B**) exposure to the test compound. TBHP at 250 μM was used as the positive control. Values are mean ± standard deviation of three independent experiments and are expressed as percentage increase in fluorescence compared to the control. Different letters indicate a significant difference (*p* < 0.05) between treatments by Tukey’s multiple comparison test.

To test the potential of OTA to induce oxidative stress in HepG2, cells were exposed to OTA concentrations ranging from 1 nM to 100 μM for 4 and 24 h, and ROS production was measured by the increase in 2’,7’-dichlorofluorescein (DCF) fluorescence (Figure 2). At both incubation times, 4 h and 24 h, OTA at 100 μM showed a significant (*p* < 0.05) increase in ROS production by 27% and 35%, respectively. In contrast, TBHP at 250 μM increased ROS production by 74% and 169% at 4 h and 24 h, respectively. These results agree with previous studies, which have shown that OTA influences ROS production levels in a dose-dependent manner [15,21,23,24,25,26,27].

### 2.3. Effect of OTA on GSH

**Figure 3:**
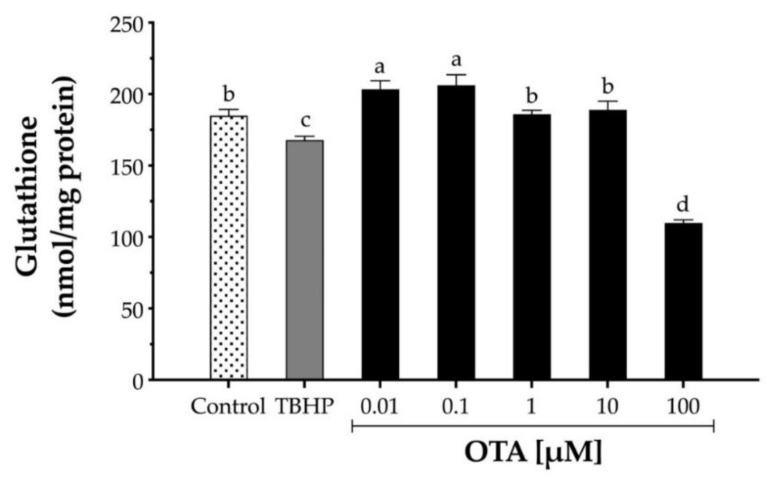
Relative levels of glutathione (GSH) in human hepatocellular carcinoma (HepG2) cells. GSH levels were determined by the reaction with 5,5′-dithiobis(2-nitrobenzoic acid) DTBN to form 5′-thio-2-nitrobenzoic acid in cell homogenates after cells were treated with TBHP and OTA for 24 h. TBHP at 1 mM was used as the positive control. Values are mean ± standard deviation of three independent experiments and are expressed as nmol glutathione per mg of protein. Different letters indicate a significant difference (*p* < 0.05) between treatments by Tukey’s multiple comparison test.

To assess the effect of OTA on GSH in HepG2, cells were exposed to increasing concentrations of OTA ranging from 10 nM to 100 μM for 24 h, and GSH levels were determined by Ellman’s method (Figure 3). While low doses of OTA (10 and 100 nM) significantly (*p* < 0.05) increased intracellular GSH levels by 9.7% and 11.3%, respectively, a higher dose of OTA (100 µM) significantly depleted GSH levels by 40.5% (Figure 3). In contrast, TBHP at 1 mM only decreased GSH by 9.2%.

### 2.4. Effect of OTA on Expression of Antioxidant Enzymes

**Figure 4:**
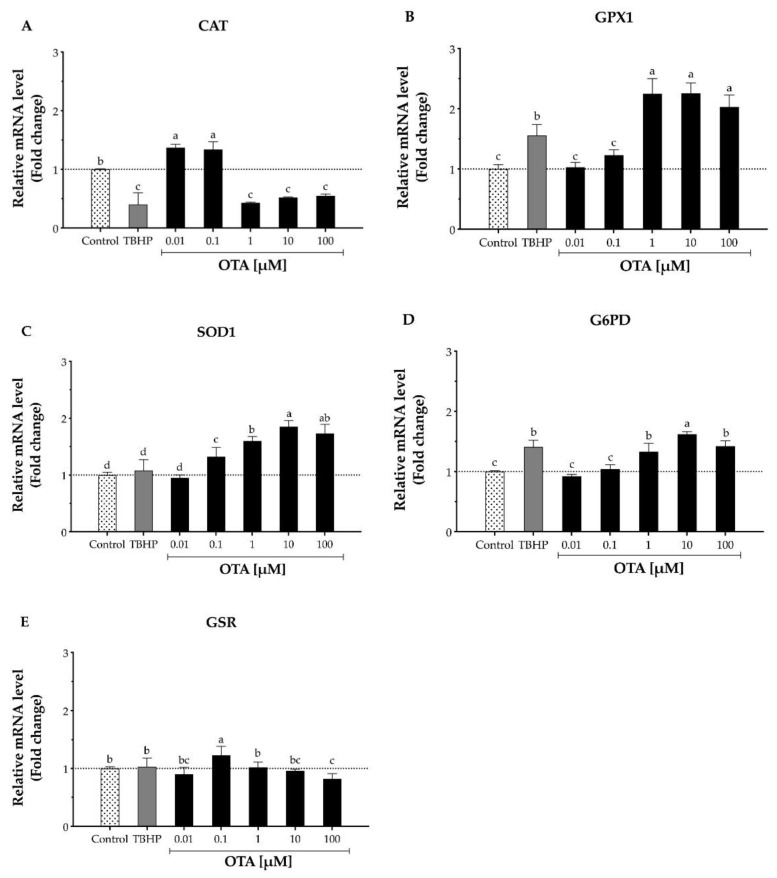
Relative mRNA levels of antioxidative enzymes in human hepatocellular carcinoma cells (HepG2) after 24 h exposure to TBHP and ochratoxin A (OTA). The mRNA expression of each gene was normalized using GADPH mRNA expression as a housekeeping gene. TBHP at 1 mM was used as the positive control. (**A**) Catalase, CAT. (**B**) Glutathione peroxidase 1, GPX1. (**C**) Superoxide dismutase 1, SOD1. (**D**) Glucose-6-phosphate dehydrogenase, G6PD. (**E**) Glutathione reductase, GSR. Values are mean ± standard error of the mean of three independent experiments and are expressed as fold change by the 2^ΔΔCt^ method. Dotted line (···) represents level of mRNA for the control cells. Different letters indicate a significant difference (*p* < 0.05) between treatments by the Student’s *t*-test.

To further understand the involvement of OTA on HepG2 cells, the mRNA levels of key antioxidant enzymes such as CAT, GPX1, SOD1, G6PD, and GSR were evaluated after 24 h OTA treatment (Figure 4). The result was considered biologically meaningful when the mRNA level changed ≥1.5-fold than the control either down- or upregulated [28]. Our results indicated that CAT mRNA levels were significantly (*p* < 0.5) downregulated upon exposure to OTA concentrations above 1 μM after 24 h, as mRNA levels ranged from 1.82- to 2.33-fold lower than the control (Figure 4A). In addition, GPX1, which is also involved in H_2_O_2_ decomposition, showed an opposite expression than CAT. After a 24 h OTA treatment, the mRNA levels of GPX1 were upregulated by 2.03- to 2.26-fold in HepG2 cells (Figure 4B). This trend was also observed on SOD1 mRNA levels, where they were upregulated by 1.60- to 1.85-fold (Figure 4C). Similar results were perceived on G6PD as the mRNA levels were upregulated from 1.62- to 1.42-fold after cells were exposed to 10 and 100 μM OTA for 24 h, respectively (Figure 4D). A high OTA dose (100 µM) downregulated the mRNA levels to 0.82-fold compared to the control (Figure 4E).

### 2.5. Effect of OTA on CAT Activity

**Figure 5:**
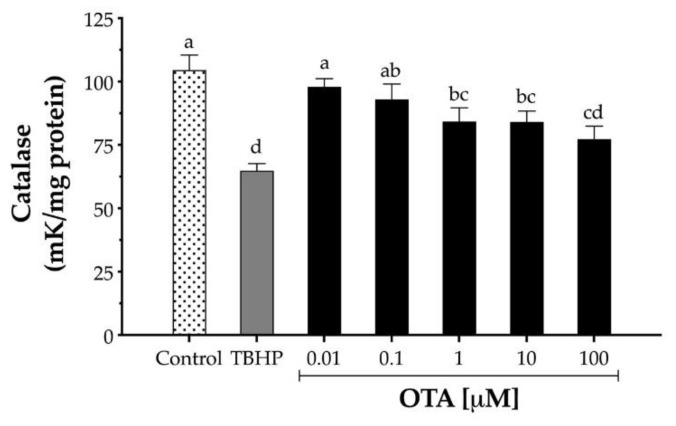
Catalase activity in human hepatocellular carcinoma cells (HepG2) after 24 h exposure to TBHP and ochratoxin A (OTA). One catalase unit was defined as the amount of catalase necessary to decompose 1 µM H_2_O_2_ per minute at pH 7.8 at 25 °C. TBHP at 1 mM was used as the positive control. Values are mean ± standard deviation of three independent experiments and are expressed as catalase units. Different letters indicate a significant difference (*p* < 0.05) between treatments by Tukey’s multiple comparison test.

Since OTA decreased the expression of CAT mRNA, the effect of OTA on CAT enzymatic activity was also evaluated. HepG2 cells were exposed to increasing concentrations of OTA (10 nM to 100 μM) for 24 h, and the CAT activity was measured by monitoring H_2_O_2_ decomposition. OTA (1 to 100 µM) significantly decreased the CAT enzymatic activity after 24 h treatment in HepG2 from to 19% to 26%, respectively (Figure 5). In contrast, cells treated with TBHP (1 mM) decreased CAT activity by 38% after 24 h exposure (*p* < 0.05).

## 3. Discussion

The exact mechanism of OTA toxicity in the liver has not been elucidated yet. According to proposed mechanisms involved in OTA toxicity, acute and chronic toxicity of OTA are related directly or indirectly to: (a) its property of inhibiting mitochondrial respiration and ATP production through competitive inhibition of transporters located in the inner mitochondrial membrane [29,30]; (b) its observed inhibition of protein synthesis by OTA, resulting from the competition with phenylalanine during the aminoacylation reaction of phenylalanine–*t*RNA [31,32]; (c) its induction of DNA damage in vivo as repeated exposure caused DNA-strand breakages in the liver of rats [8,33]; (d) its capacity to induce lipid peroxidation [34,35]; and (e) the production of ROS and resulting oxidative stress observed in several in vitro and in vivo experiments [17,36,37]. Based on the toxicological data available to date, oxidative stress appears to be the most plausible underlying mechanism of OTA toxicity [38,39]. However, the involvement of oxidative stress in OTA-mediated toxicity in liver remains debatable.

It is known that the presence of serum in the culture medium influences OTA-induced cytotoxicity due to OTA ability to bind to serum proteins, and thus, lowering OTA cytotoxicity; however, in agreement with our results, previous studies have observed the cell viability plateau above 1 μM OTA, in both serum-free and serum-containing growth medium [15,22,40]. In addition, this effect has also been observed in both the human hepatocellular carcinoma cell line (Hep3B) and in primary cultured human hepatocytes, when OTA concentrations tested were above 10 and 5 μM OTA, respectively [41,42]. A hypothesis behind this observation has suggested the existence of an OTA carrier system being saturated at low OTA-concentrations; consequently, OTA uptake by the cell is limited [40]. In rat hepatocytes, an organic anion-transporting polypeptide (*oatp*) has been shown to be involved in OTA uptake [43]. However, as the plateau in cell viability has been observed in both serum-free and serum-containing medium, another hypothesis that may explain the survival of HepG2 cells at higher OTA-concentration exposure is related to OTA solubility in water. OTA solubility in water at 25 °C has been reported to be between 1.05 and 3.25 μM, and thus, OTA could not be dosed high enough to reach a maximal cytotoxic effect due to its solubility [44,45].

ROS are produced by the cell during normal aerobic metabolism and possess a dual function on the cell. While lower ROS levels are required for several physiological mechanisms such as host defense [46], excessive levels of ROS negatively affect cell viability via lipid peroxidation and protein and nucleic acid oxidation [47]. Several studies observed increased/overproduced free radicals by exposing OTA in both in vitro and in vivo [17,20,36,37,48,49,50] and it has been linked to OTA toxicity. Although our results suggest that OTA can cause an increased ROS production at higher doses (100 µM), the toxicity observed at lower doses (1 and 10 µM) could not be explained by ROS production. GSH is an important intracellular antioxidant molecule that reacts directly against ROS and indirectly as a cofactor for antioxidant enzymes. Our results showed that OTA at lower doses (0.01 and 0.1 µM) increased the levels of intracellular GSH, whereas a higher OTA dose (100 µM) significantly decreased GSH levels, which agrees with previous studies on HepG2 [15,40,51]. A reduction in GSH after rat primary hepatocytes were exposed to OTA was associated with a decrease in the expression of glutamate cysteine ligase; the rate-limiting enzyme in GSH synthesis [36]. In addition, it has been proposed that GSH is involved in OTA detoxification through direct binding [52]. Thus, our results suggest that high OTA doses may reduce the intracellular GSH pool either through direct binding and/or interfering with de novo synthesis of GSH.

While an in vitro approach is often used to study ROS production/reduction to determine cytotoxicity, Zhu et al. [53] reported that there was no evident dose-response relationship between ROS and cytotoxicity. Several theories can be considered to explain our results. A concentration-independent evaluation of ROS level may be explained because of a loss in the mitochondrial membrane potential, and thus, once cytochrome *c* is released from the mitochondria, it may react with H_2_DCFDA, increasing DFC fluorescence [24,54]. It is also possible that peroxidases can activate the step and produce the OTA phenoxyl radical [55], which may be converted back to OTA in the presence of GSH. However, such a conversion would require the formation of superoxide anion radical (O_2_^•−^) [56]. As a Fenton reaction can be induced by SOD catalyzed conversion of O_2_^•−^ to H_2_O_2_, hydroxide radical (HO•) may lead to oxidative damage. This mechanism may corroborate depletion of GSH by OTA in cellular models [8]. In addition, the phenoxyl radical is known to form C_8_-deoxyguanosine adducts [57].

Mammalian cells handle ROS mainly through three crucial types of antioxidant enzymes: CAT, SOD, and GPX. We observed that OTA modulates the gene expression of these antioxidant enzymes differently. First, OTA had a binary effect on CAT, lower OTA doses (0.01 and 1 µM) upregulated CAT expression, while OTA concentrations above 1 µM downregulated it; second, OTA above 0.1 µM upregulated the expression of SOD1 in a dose-dependent manner; and third, OTA above 1 µM upregulated the expression of GPX1. Accordingly, our results agreed with previous in vitro studies with HepG2 and Vero (kidney) cells where OTA at 25 µM showed a reduction of CAT and an increase in SOD1 gene expression [15,16,23]. It is important to mention that GPX requires secondary enzymes (G6PD and GSR) and cofactors (GSH, NADPH, and glucose-6-phosphate) to function efficiently. In addition, we observed that OTA above 1 µM upregulated the expression of G6PD, suggesting that the pool of glucose-6-phospate and NADPH was not impaired. Furthermore, we noticed that OTA at higher doses (100 µM) downregulated the expression of GSR, which may explain the decrease in intracellular GSH noted as GSR is required to reduce glutathione disulfide to regenerate GSH [18]. Moreover, we also saw a reduction in CAT enzymatic activity as increasing OTA doses, which agreed with previous studies on HepG2 and Vero cells upon 10 µM OTA treatment after 24 h and 48 h [15,16]. Thus, our results indicate that the decrease in CAT activity may be linked to the downregulation of CAT gene expression.

## 4. Conclusions

The most important finding about this study was that OTA is cytotoxic to HepG2 cells in a time-dependent manner, which could not be directly associated with an increase in ROS production, but a depletion of GSH and modulation on the mRNA expression of antioxidant enzymes and its activity. OTA significantly downregulated the gene expression of CAT, which was concomitant to a decrease in its enzymatic activity. Therefore, the cells rely on the GPX system for the removal of H_2_O_2_. Although the cell upregulated the expression of GPX1 after OTA exposure, it also decreased the expression of GSR, which led to an impairment of H_2_O_2_ detoxification by the glutathione peroxidase system. Additionally, it is noteworthy to mention that OTA mediated cytotoxicity in HepG2 cells did not follow a clear dose-response relationship, and for the first time, we have indicated that the maximum reported OTA solubility in water is 3 µM; which explains the plateau observed at higher OTA doses. In conclusion, the results presented in this study indicate that OTA may be involved in the development of oxidative stress through increasing intracellular H_2_O_2_ levels by impairing the primary antioxidant enzymes catalase and glutathione peroxidase.

## 5. Materials and Methods

### 5.1. Chemicals

Ochratoxin A (OTA), 3-(4,5-Dimethyl-2-thiazolyl)-2,5-diphenyl-2H-tetrazolium bromide (MTT), sodium dodecyl sulfate (SDS), N,N-dimethylformamide (DMF), reduced L-glutathione reduced (GSH), *tert*-butyl hydroperoxide (TBHP), hydrogen peroxide (H_2_O_2_), protease inhibitor cocktail, catalase from bovine liver (≥10,000 U/mg protein), sodium chloride, potassium chloride, magnesium chloride, calcium chloride, glucose, *N*-(2-hydroxyethyl)piperazine-2′-(2-ethane-sulfonic acid) (HEPES), and 5,5′-dithio-bis(2-nitrobenzoic acid) (DTNB) were purchased from Sigma-Aldrich (St. Louis, MO, USA). Eagle’s minimum essential medium (EMEM), penicillin-streptomycin (10,000 U/mL–10,000 μg/mL), Dulbecco’s phosphate-buffer (no calcium and magnesium, DPBS), fetal bovine serum (FBS), 0.25% *w*/*v* trypsin-0.53 mM EDTA, 6-carboxy-2’,7’-dichlorodihydrofluorescein diacetate (carboxy-H_2_DCFDA), M-PER mammalian protein extraction reagent, Pierce Bicinchoninic Acid Protein Assay Kit (BCA), TRIzol reagent, Quant-iT RiboGreen RNA Assay Kit, and High-Capacity cDNA Reverse Transcription Kit were obtained from Invitrogen (Waltham, MA, USA). RNeasy Plus Mini Kit was purchased from Qiagen (Germantown, MD, USA).

### 5.2. Cell Culture

Human hepatocellular carcinoma derived cell line (HepG2) was obtained from the American Type Culture Collection (ATCC) and cultivated according to ATCC culture method recommendations. HepG2 cells were cultured in EMEM medium containing 1% (*v*/*v*) penicillin-streptomycin and 10% (*v*/*v*) FBS. Cells maintained at 37 °C in a humidified atmosphere with 5% CO_2_. HepG2 cells were seeded in each specific assay plate at a density of 1.0 × 10^5^ cells/cm^2^. OTA was dissolved in pure methanol (24.76 mM) and further diluted in EMEM under serum-free conditions; methanol concentration did not exceed 0.1% (*v*/*v*) in the final assay.

### 5.3. Cell Viability Assay

Cell viability was determined using the MTT assay, which is a widely accepted method to enumerate viable cells [20]. Briefly, cells were seeded in 96-well plate and allowed to grow for 24 h, and then treated with increasing concentrations of OTA (1 nM to 100 μM) in serum-free EMEM for different time intervals (24, 48, and 72 h). After OTA exposure, MTT was added directly to the medium (final concentration of 0.5 mg/mL) and the plate was incubated for 4 h at 37 °C. The formazan crystals formed were dissolved with 20% *w*/*v* SDS in DMF (pH 4.7), and the absorbance was read at 570 nm on a microplate reader (SpectraMax 190, Molecular Devices, Sunnyvale, CA, USA). The cell viability was calculated with respect to non-treated cells as the control, and thus the results were expressed as percentage of control. The percentage of cell viability was calculated by the following formula: % cell viability = (*Abs_sample_* − *Abs_blank_*)/(*Abs_control_* − *Abs_blank_*) × 100, where *Abs* = absorbance value.

### 5.4. ROS Analysis

The redox state of the cells was assessed using carboxy-H_2_DCFDA conversion to fluorescent 2′,7′-dichlorofluorescein (DCF) [37]. Briefly, cells were seeded in black and clear bottom 96-well plate in EMEM and allowed to grow for 24 h, the medium was removed, and cells washed with Krebs–Ringer (KRH) buffer (116 mM NaCl, 4 mM KCl, 1 mM MgCl_2_, 1.8 mM CaCl_2_, 25 mM glucose, and 10 mM HEPES, pH 7.4). Next, cells were incubated with 100 μM carboxy-H_2_DCFDA in KRH buffer for 30 min at 37 °C. Carboxy-H_2_DCFDA was removed by washing the cells once with KRH buffer, and then treated with increasing concentrations of OTA (1 μM to 100 μM). The fluorescent of the cells from each well was measured and recorded after 4 h and 24 h in a Sinergy 2 microplate reader (BioTek, Winooski, VT, USA) with a temperature maintained at 37 °C. The excitation filter was set at 485 ± 20 nm and the emission filter was set at 528 ± 20 nm. Tert-butyl hydroperoxide (TBHP) was used as the positive control for ROS. The results were expressed as percentage of control, which was calculated by the following formula: ROS (% of control) = (*F_sample_* − *F_blank_*)/(*Fc_ontrol_* − *F_blank_*) × 100, where *F* = fluorescence value.

### 5.5. Intracellular GSH Analysis

Reduced glutathione was determined spectrophotometrically by quantifying the oxidation product (5′-thio-2-nitrobenzoic acid) upon the reaction between GSH and DTBN [52]. Briefly, cells were seeded in 6-well culture plates and allowed to grow for 24 h, culture medium was removed, and then cells were exposed to increasing concentrations of OTA (10 nM to 100 μM) and 1 mM TBHP in serum-free EMEM for 24 h. Then, cells were harvested by trypsinization and washed with DPBS. Cell pellets was lysed with M-PER supplemented with 1% (*v*/*v*) protease inhibitor cocktail, and after 5 min incubation, it was homogenized with a Teflon pestle for 30 s, followed by centrifugation at 12,000× *g* for 10 min at 4 °C. All assays were carried out in triplicate and the results were expressed as nmol GSH/mg protein. Glutathione concentrations were calculated based on a standard curve of reduced glutathione using linear regression, and protein content was determined by the Pierce BCA protein assay.

### 5.6. Quantitative Real-Time Polymerase Chain Reaction (PCR)

The relative expression of mRNA levels of selected genes encoding antioxidant enzymes was assessed by TaqMan gene expression assays. Cells were seeded in 100 mm culture dishes and allowed to grow for 24 h; afterward, cells were exposed to increasing concentrations of OTA (10 nM to 100 μM) in serum-free EMEM for 24 h. Cells were then washed with DPBS, and RNA was extracted using the TRIzol reagent following the manufacturer’s instructions. Total RNA was purified using RNeasy Plus Mini Kit according to the manufacturer’s procedure. Total RNA was quantified fluorometrically using the Quant-iT RiboGreen RNA Assay Kit according to the manufacturer’s procedure. First-strand cDNA was synthesized from 2 μg of RNA using the High-Capacity cDNA Reverse Transcription Kit according to the manufacturer’s procedure, and 10 ng of cDNA for gene expression analysis was used. TaqMan gene expression master mix with pre-validated TaqMan gene expression assays were SOD1 (Hs00533490_m1), CAT (Hs00156308_m1), GPX1 (Hs00829989_gH), GSR (Hs00167317_m1), G6PD (Hs00166169_m1), and GAPDH (Hs99999905_m1). All assays were performed in triplicate and cycle threshold (CΤ) values were used for further analysis. The relative constitutive gene expression was calculated by the ΔCΤ method using a StepOne Plus real-time PCR system (Applied Biosystems, Foster City, CA, USA). Changes in gene expression in the groups treated with OTA were calculated by the ^ΔΔCΤ^ method [53]. CΤ values were normalized to the housekeeping gene GAPDH.

### 5.7. Catalase Activity Analysis

Cells were seeded in 6-well plates and treated as described in Section 2.5. and CAT activity was determined as described elsewhere [54]. All assays were performed in triplicate and specific activities expressed as units per mg of protein. TBHP at 1 mM was used as a positive control. One CAT unit was defined as the amount of enzyme necessary to decompose 1 μM of H_2_O_2_ per minute at pH 7.8 at 25 °C. Hydrogen peroxide decomposition was monitored at 240 nm using a Synergy 2 microplate reader (BioTek, Winooski, VT, USA).

### 5.8. Statistical Analysis

Statistical calculations were performed using the Statistical Package for Social Sciences version 23.0 (SPSS Inc., Chicago, IL, USA). The effect of different concentrations of OTA on individual parameters was analyzed by analysis of variance (ANOVA) and Dunnett’s test, and multiple comparisons of means was done with Tukey’s test or with the Student’s *t*-test for two group comparisons. Values represent means ± standard deviation (SD) of three independent experiments and subsequent cell passages. Differences were accepted as statistically significant at *p* < 0.05.

## Data Availability

The data presented in this study are available in article here.
